# Automated radiosynthesis of [^11^C]UCB‐J for imaging synaptic density by positron emission tomography

**DOI:** 10.1002/jlcr.3828

**Published:** 2020-02-06

**Authors:** Selena Milicevic Sephton, Tunde Miklovicz, Joseph J. Russell, Aniruddha Doke, Lei Li, Istvan Boros, Franklin I. Aigbirhio

**Affiliations:** ^1^ Radiopharmaceutical Unit, Wolfson Brain Imaging Centre, Department of Clinical Neurosciences, School of Clinical Medicine University of Cambridge Cambridge UK; ^2^ University of Debrecen, Faculty of Medicine Department of Medical Imaging, Division of Nuclear Medicine and Translational Imaging, Department of Medical Imaging, Faculty of Medicine H‐4032 Nagyerdei krt. 98 University of Debrecen Debrecen Hungary

**Keywords:** [^11^C]UCB‐J, carbon‐11 radiosynthesis, Synthra RNPlus, SV2A protein, synaptic density, Suzuki cross‐coupling

## Abstract

An automated radiosynthesis of carbon‐11 positron emission tomography radiotracer [^11^C]UCB‐J for imaging the synaptic density biomarker synaptic vesicle glycoprotein SV2A was established using Synthra RNPlus synthesizer. Commercially available trifluoroborate UCB‐J analogue was used as a radiolabelling precursor, and the desired radiolabelled product was isolated in 11 ± 2% (n = 7) nondecay corrected radiochemical yield and formulated as a 10% EtOH solution in saline with molar activities of 20 to 100 GBq/μmol. The method was based upon the palladium(0)‐mediated Suzuki cross‐coupling reaction and [^11^C]CH_3_I as a radiolabelling synthon. The isolated product was cGMP compliant as demonstrated by the results of quality control analysis.

**Abbreviations**
PET: positron emission tomography
SV2A: synaptic vesicle glycoprotein 2A
LCMS: liquid chromatography mass spectrometry
HPLC: high‐pressure liquid chromatography
TLC: thin‐layer chromatography

## 1 INTRODUCTION

Synapses are specialized structures in the brain, which mediate a functional interaction between two neurons or between a neuron and another cell type.^1^ They are involved in processes of higher brain functions such as information processing (eg, thinking) or changes with experience (eg, learning). As such, synapses are targets of many psychoactive drugs and can become defective in neurological diseases as well as psychiatric disorders.^2^, ^3^, ^4^, ^5^, ^6^ While the synaptic density can be used as a quantitative biomarker of synaptic pathology, in human subjects, this can only be determined through a post mortem autopsy or surgical resection.^7^

**Figure 1:**
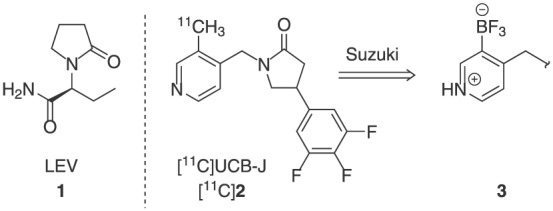
Positron emission tomography radiotracers for imaging SV2A, a biomarker of synaptic density and the radiosynthetic strategy used for preparation of [^11^C]UCB‐J

Recently developed is a noninvasive alternative using positron emission tomography (PET), which for the first time enables the quantification of synaptic density in vivo. This is achieved by radiotracers selective to synaptic vesicle glycoprotein SV2A, a transmembrane protein expressed ubiquitously in secretory vesicles in all brain areas.^8^ Arising from several structural scaffolds related to the antiepileptic drug levetiracetam (LEV, **1**; Figure 1)^9^ the carbon‐11 radiolabelled UCB‐J analogue ([^11^C]**2**; Figure 1) has been shown to have excellent in vitro and in vivo properties for imaging SV2A from both preclinical and clinical studies.^7^, ^10^

Therefore, to undertake clinical SV2A PET studies at our centre, our objective was to establish a manufacturing method for [^11^C]UCB‐J for which a radiosynthesis method has been developed.^7^, ^10^, ^11^ Herein, we report development of the fully automated and cGMP compliant radiosynthesis of [^11^C]UCB‐J using Synthra RNPlus automated radiosynthesizer.

## 2 MATERIALS AND METHOD

### 2.1 General techniques

All anhydrous solvents were purchased from Aldrich, Alfa Aesar, or Acros and used as received unless otherwise noted. Water for injection was purchased from Braun and sodium chloride 0.9% w/v injection BP from Kent Pharmaceuticals. Water for cleaning of the synthesizer was Millipore (18.2 MΩ), and water used for the radiosynthesis was BP grade. The radiolabelling precursor and the reference material were obtained from UCB, Belgium. The Sep‐Pak Classic C18 cartridges were purchased from Waters and the empty 3‐mL SPE columns with frits Chromabond from Macherey‐Nagel. Ethanol for injection was purchased from Merck.

Radiochemical yields for the product were based on [^11^C]CH_3_I and decay corrected unless otherwise stated.

### 2.2 Radiosynthesis of [^11^C]UCB‐J on Synthra RNPlus

Prior to each production, the automated synthesizer was cleaned using H_2_O and absolute EtOH and acetone. The [^11^C]CH_3_I delivery line was tested for flow and leak tightness. The GE MeI module was preconditioned (heating followed by the cooling of the Ni‐ and porapak ovens) using automated PREPARATION stage of the module.

**Figure 2:**
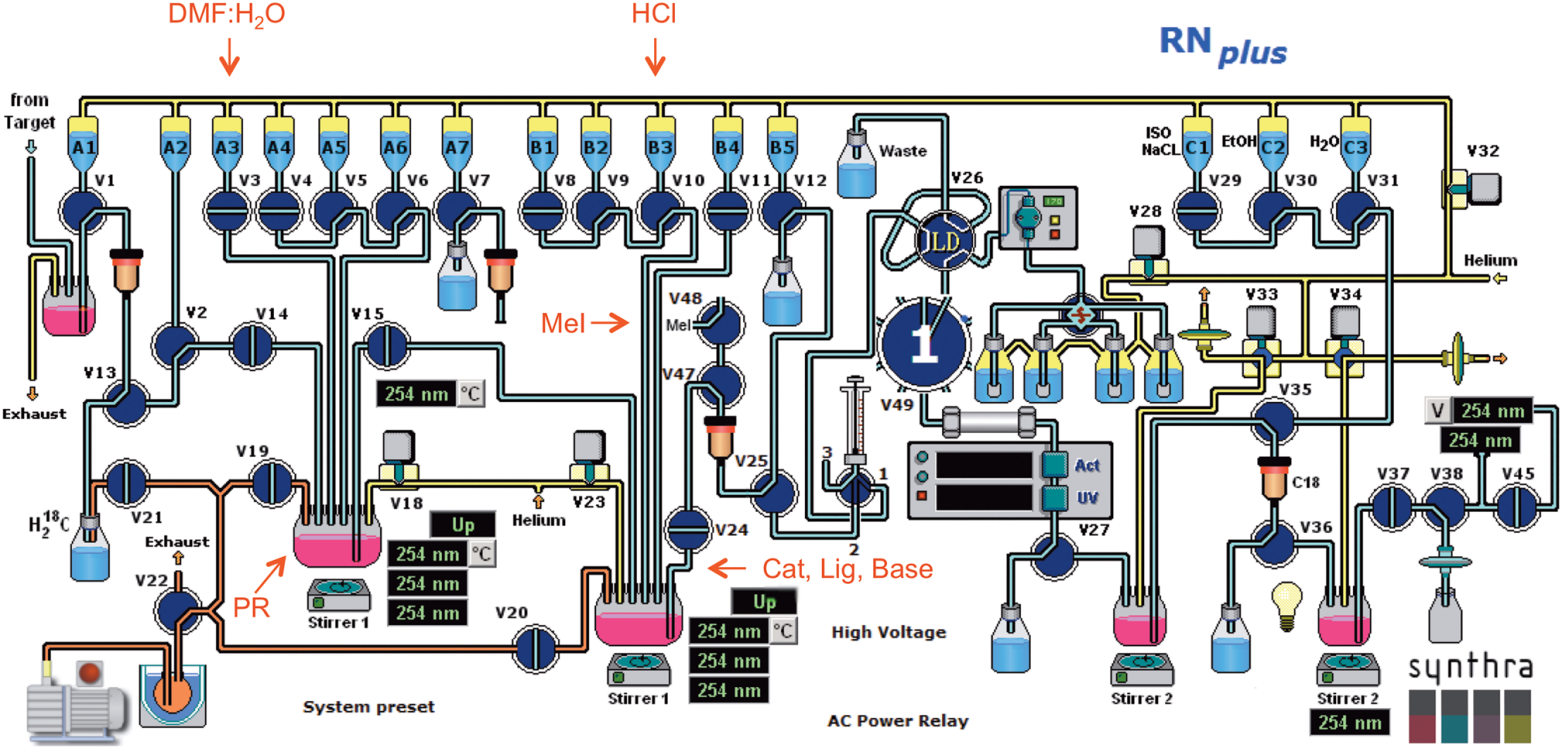
Automated radiosynthesizer Synthra RNPlus as the platform for the radiosynthesis of [^11^C]UCB‐J

The automated Synthra RNPlus set up was as follows (see Figure 2):

**A3**: 0.3 mL DMF:H_2_O 8:1 v/v
**B3**: 1.6 mL 1M aq HCl
**C1**: 13 mL NaCl 0.9% BP
**C2**: 1.4 mL absolute EtOH
**C3**: 15 mL H_2_O for injection
**SPE**: 50 mL H_2_O for injection
**C18**: Classic cartridge, conditioned with 5 mL EtOH, 10 mL H_2_O, then 2 mL air
**Filter frit**: filled with 3.2 mL HPLC eluent, placed between V47 and V25
**HPLC**: Semipreparative column Gemini‐NX C18 250x10 mm, 5 μm, 100 Å, eluting with 65% 100 mM ammonium formate pH 9.2 and 35% MeCN; flow 8 mL/min, λ = 254 nm
**REACTOR 1**: 1.6‐1.7 mg (*R*)‐trifluoro(4‐((2‐oxo‐4‐(3,4,5‐trifluorophenyl)pyrrolidin‐1‐yl)methyl)pyridin‐1‐ium‐3‐yl)borate (UCB‐J precursor) in 54 μL MeOH and 22 μL 1M aq HCl
**REACTOR 2**: 28 μL potassium carbonate aqueous solution (4‐5 mg in 280 μL H_2_O), 244 μL bis (dibenzylideneacetone)palladium(0) solution in anhydrous DMF (1.0‐1.5 mg Pd (dba)_2_ in 488 μL DMF), 200 μL tris(*o*‐tolyl)phosphine solution in anhydrous DMF (2.0‐2.5 mg P(*o*‐Tol)_3_ in 500 μL DMF), and 500 μL anhydrous DMF
**PRECURSOR ACTIVATION**: Precursor activation is a step that commences at the same time as the cyclotron target bombardment starts and precedes the oxidative insertion of Pd to carbon‐halide bond. The activation of the precursor is achieved by placing the precursor into the reactor 1, adding MeOH and HCl, and stirring the mixture over 30 minutes at ambient temperature. The mixture is then dried in the stream of He and under vacuum and finally only vacuum after which it is redissolved in DMF:H_2_O (0.3 mL, 8:1 v/v) and transferred from reactor 1 to reactor 2.

[^11^C]CO_2_ is produced *via* the nuclear reaction ^14^N(p,α)^11^C using GE PETtrace cyclotron from 0.5% O_2_ enriched N_2_. Typically bombardment was performed at 35 μAh over 40 to 45 minutes. The produced [^11^C]CO_2_ was then converted to [^11^C]CH_3_I using a GE MeI module via reduction to [^11^C]CH_4_ using a Shimalite nickel catalyst/column followed by condensation reaction of [^11^C]CH_4_ with I_2_ at 720°C during the recirculation after which [^11^C]CH_3_I is trapped on the Porapak and released upon heating. The produced [^11^C]CH_3_I was received in the reactor 2 of the Synthra RNPlus automated module and bubbled through the mixture containing base, ligand, and catalyst solution and DMF (see **REACTOR 2**) at –15°C for 4 to 7 minutes during the number of counts per second in the reactor 2 peaked. The reaction mixture was then heated to 30°C for 3 minutes to allow for oxidative insertion of Pd to carbon‐halogen bond to occur. The activated precursor was then added (see **PRECURSOR ACTIVATION**) as DMF:H_2_O solution, and the reaction mixture was allowed to heat to 100°C over 5 minutes. After this time, the reaction mixture is allowed to cool to 30°C and then quenched with HCl from B3 and loaded onto the high‐pressure liquid chromatography (HPLC) loop via the syringe and by passing the mixture through the SPE column with frit containing eluent. The semipreparative purification finally yields desired [^11^C]UCB‐J, which is collected in the SPE flask containing H_2_O and then trapped on the C18 Classic cartridge. The product is washed with H_2_O and then eluted with EtOH and formulated as a saline solution and dispensed in the grade A isolator by passing the mixture through the sterile filter.

### 2.3 QC analysis

Prerelease QC of [^11^C]UCB‐J took approximately 30 minutes and was performed as described previously.^12^ Release criteria and the results of three typical production experiments are summarized in Table 2. The pH of the product was determined using Seven Excellence pH meter, and the visual check was performed behind a lead‐shielded window. Chemical and radiochemical identity and purity were analysed by analytical radio‐HPLC on the ThermoFisher Ultimate 3000 HPLC system using Waters XBridge C18 reverse phase column (3.5 μm, 100×3.0 mm) eluting with 30% MeCN in 10 mM sodium phosphate at 0.8 mL/min. The HPLC system was equipped with an UV detector (λ = 258 nm) and EG&G Ortec Amplifier 0.70 kV radioactivity detector. The radiochemical purity was determined as the percentage of the [^11^C]UCB‐J peak on the radio‐chromatogram. Radiochemical identity was confirmed and assessed by comparison of the retention time of the [^11^C]UCB‐J radioactive peak to that of the UCB‐J reference standard peak in the coinjection sample. The chemical amount of [^11^C]UCB‐J and cold impurities was determined by quantification and relative comparison of the corresponding UV absorbance peaks of the QC sample and reference standards of known concentration. The acceptance limit of 10 μg for UCB‐J in the injection dose was established based on the selected animal toxicity study taking into account guideline on setting permitted daily exposure by European Medical Agency. The maximal allowable injection volume (*V*_max_) of radiotracers produced at the Wolfson Brain Imaging Centre (WBIC) is by default limited to 10 mL and was calculated based on the chemical amount of [^11^C]UCB‐J and cold impurities relative to their specification *V*_max_ = 10 μg/specification of [^11^C]UCB‐J found in a QC sample (μg/mL). Molar activity (*A*_*m*_) was calculated as the ratio of radioactivity in GBq and the amount of UCB‐J (μmol) at the time of anticipated application (20 min after the EOS). The integrity of the Acrodisc Supor Membrane filter (0.2 μm, 25 mm) was tested using the filter integrity (ie, bubble point) test. Sterility, bacterial endotoxins, radionuclidic identity, and residual solvent analyses of the product were assessed as the postrelease tests. Endotoxin content in [^11^C]UCB‐J doses was measured with the Charles River Laboratories Endosafe Portable Test System. Residual solvent analysis was performed on Thermo Focus Gas Chromatograph equipped with the Thermo column (6′×1.8′×0.85″) and a flame ionization detector. Radionuclidic identity was determined by measuring [^11^C]UCB‐J activities in triplicate at different time points (5‐min intervals) using Veenstra VDC‐505 radioisotope dose calibrator, and the half‐life was calculated as per the following: *t*_1/2_ = –ln2·((*t*_2_ – *t*_1_)/ln(*A*_2_/*A*_1_)), whereby *t*_1_ and *t*_2_ are two different times and *A*_1_ and *A*_2_ are radioactivity measured at times *t*_1_ and *t*_2_, respectively.

## 3 RESULTS AND DISCUSSION

**Scheme 1:**
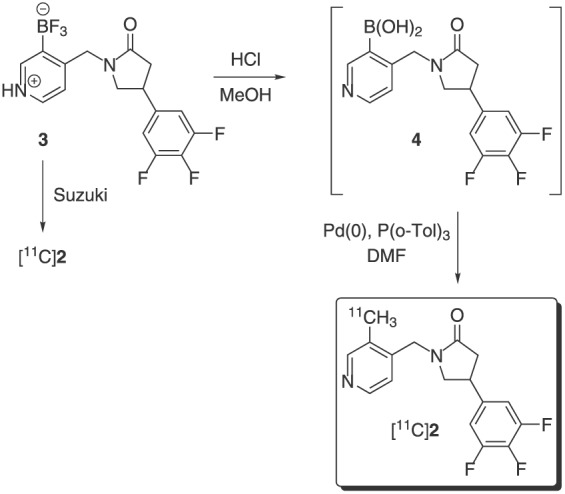
Radiosyntheses of [^11^C]UCB‐J via direct Suzuki cross‐coupling of borate precursor **3** or *via* the boronic acid intermediate

The radiosynthesis of [^11^C]UCB‐J is based on the application of the palladium(0)‐mediated Suzuki cross‐coupling reaction between the carbon‐11 methyliodide ([^11^C]CH_3_I) and trifluoroborate precursor **3** (Figure 1).^7^, ^10^, ^11^, ^13^, ^14^ Initially explored method employed a more common Suzuki reacting partner, ie, the corresponding boronic acid^15^ (eg, **4** in Scheme 1) as a radiolabelling precursor; however, the boronic acid precursor **4** proved to be unstable upon storage resulting in poor reproducibility and unreliable radiochemical yields. Further investigation indicated that while trifluoroborate **3** can be used as a radiolabelling precursor for [^11^C]UCB‐J, the actual reacting specie is the boronic acid, thus necessitating a need for a mixture of trifluoroborate **3** and 3‐10% of the boronic acid **4** to be present in the reaction vessel.^11^, ^14^

While the application of the mixture improved reproducibility of the method, it made it non‐GMP compliant for which reason Onega and coworkers^11^ developed an improved protocol whereby the boronic acid was formed in situ by treatment of trifluoroborate **3** with 1M HCl (Scheme 1).

In our hands, in situ preparation of boronic acid worked sluggishly (Table 1 and Table S1). Based on the method developed by Onega et al,^11^, ^14^ hydrochloric acid was employed and reaction progress monitored by the liquid chromatography‐mass spectrometry (LCMS) using HCl in different solvents. The application of HCl in dioxane gave none of the desired boronic acid **4** (entry 1). In methanolic HCl, a 24% conversion by LCMS was achieved after 40 minutes (entry 2). Using the aqueous solution of HCl (c=1 M) afforded desired **4** in maximum 39% LCMS conversion after repeated addition of the acid and extended reaction time. We speculated that it was the concentration of the reaction mixture, which determined the conversion to the desired boronic acid **4**. To test this, the aqueous HCl used by Onega et al^11^ was employed at 63 and 6.3 mM total reaction concentrations to afford 0% and 38% LCMS conversion over the same period of time, respectively. This suggested that complete conversion to boronic acid was not essential; however, the mixture of unreacted trifluoroborate **3** and boronic acid **4** was required to be free from MeOH/HCl prior to Suzuki cross‐coupling reaction. The smaller volume of solvents (MeOH/HCl) would be quicker to remove; thus, we set out to use 63 mM concentration and perform the radiochemical step next. To implement an automated method for [^11^C]UCB‐J manufacture, we decided to use a Synthra RNPlus module (Figure 2).

**Table 1:** Various conditions for the in situ preparation of boronic acid precursor **4**

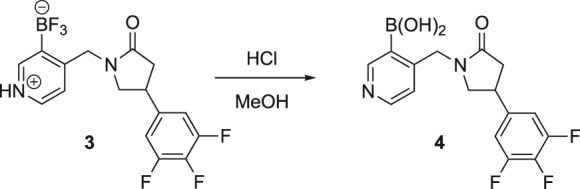				
Entry	Conditions	Concentration, mg 3 in HCl: MeOH	Time, min	LCMS Conversion, %
1	HCl in dioxane	2 mg in 0.25:2 mL	40	0
2	HCl in MeOH	3 mg in 0.5:3 mL	40	24
3	HCl in H_2_O	1.9 mg in 0.5:2 mL	190	39^a^
**4**	**HCl in H**_**2**_ **O**	**1.8 mg in 22:54 μL**	**32**	**0**
5	HCl in H_2_O	1.8 mg in 0.2:0.5 mL	37	38^b^

a Aq. HCl added in 2×0.25 mL portions.

b Reaction performed with the crude material.

While the main disadvantage of using Synthra RNPlus module, which is intended for radioactive fluorinations, was a requirement for the external source of [^11^C]CH_3_I, the module presented several advantages particular to the case of [^11^C]UCB‐J as follows: (a) two reactors that are ideal for the in situ preparation of the reacting precursor **3**/**4** and subsequent Pd‐mediated Suzuki coupling; (b) size of the reactors which allows for larger volumes particularly for the injection of crude reaction mixture onto semipreparative HPLC column; spare valves that allow for introduction of external gases, eg, [^11^C]CH_3_I; (d) spare connection points for the filter frit and the SPE cartridge to allow filtration of the crude mixture and product formulation; and (e) easily replaceable HPLC injection loop and corresponding injection syringe to allow for 5‐mL injection volumes.

**Scheme 2:**
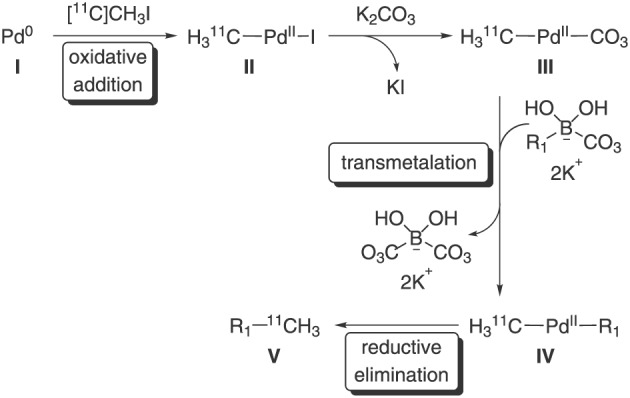
Proposed mechanism of Pd‐mediated Suzuki cross‐coupling reaction to obtain [^11^C]UCB‐J

The availability of two reactors proved beneficial for two reasons. It was quickly determined that the activation/hydrolysis of precursor **3** cannot be performed in the same reactor as the subsequent coupling, presumably due to the deactivation of Pd‐phosphine complex in the presence of acid. Furthermore, considering the mechanism of classical Suzuki cross‐coupling reaction (Scheme 2), Pd‐complex insertion into the carbon‐halide bond of [^11^C]CH_3_I (**I** to **II**), ie, oxidative addition, must precede transmetallation (**III** to **IV**) for which reason the activated precursor **3**/**4** is added once the [^11^C]CH_3_I trapping is complete and reactants allowed time for oxidative insertion. Allowing the oxidative insertion step to occur ahead of the addition of boron analogue enabled the successful conversion to the product as has been demonstrated previously for other Suzuki transformations.^16^

**Table 2:** Critical parameters in the radiosynthesis of [^11^C]UCB‐J as determined through repeated experiments (n = 54)

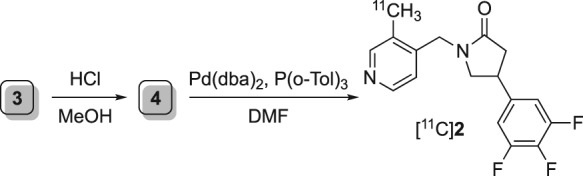		
Entry	Parameter/Step	Improvements in Reproducibility Achieved When
1	Drying activated precursor	Drying performed with vacuum in the stream of He
2	Reaction solvent DMF	Dried over molecular sieves. Bottle replaced every 2 weeks
3	Trapping of [^11^C]CH_3_I	Reaction volume in reactor 2 increased to 1.1 mL and reactor 2 pressurised before trapping
4	Semi‐prep HPLC purification	Using 5 mL injection loop and diluting the reaction mixture with eluent and filtering prior to injection
5	Quality of [^11^C]CH_3_I	Using [^11^C]CH_3_I with high molar activity

In an initial radiosynthesis attempt, 35% of the [^11^C]CH_3_I was converted to [^11^C]UCB‐J as assessed by radio‐HPLC using the following setup: reactor 1 was charged with the precursor **3**/**4**, MeOH and 1M aq HCl; reactor 2 was charged with Pd(0)‐catalyst, (*o*‐Tol)_3_P and the base K_2_CO_3_ (Figure 2). After 30 minutes of acid‐promoted hydrolysis of **3**, the activated precursor **3**/**4** was dried in the stream of He over 10 minutes. At the same time, freshly produced [^11^C]CH_3_I was trapped in the mixture of catalyst, ligand, and base in total volume of 0.5 mL and then heated to 30 °C over 3 minutes after which time activated **3**/**4** in DMSO was added and reaction proceeded at 130°C. In a repeated follow‐up attempt, 0% conversion to [^11^C]UCB‐J was observed. This discrepancy in the observed conversion to the product led to the detailed investigation of reaction parameters and helped identify critical aspects of the radiosynthesis (Table 2).

The most important factor determining the success of the reaction proved to be efficient removal of HCl/MeOH from the activated precursor **3**/**4**. Thus, solvent removal using a stream of helium only gave variable results for which reason solvent removal under vacuum in the stream of helium was employed. Using the material activated following this method warranted reliable results in the subsequent cross‐coupling step. DMF is a hygroscopic solvent, and reliable radiosynthesis of [^11^C]UCB‐J was achieved if the bottle was replaced every 2 weeks. While the exact reasons for this requirement remain unclear, it was proposed that the moisture from the DMF could affect the reaction success. Next important parameter, which improved the trapping efficiency, was the volume of the reaction mixture. Having a large 10‐mL reactor required the total volume of the reaction mixture to be increased to 1.1 mL (from 0.5 mL previously reported by others). The reliable radiosynthesis was achieved however, only if the reactor was pressurized immediately after the [^11^C]CH_3_I trapping, significantly limiting the radioactivity losses (Table 2). Another varying factor was reliable loading of the crude reaction mixture onto the HPLC loop. The crude reaction mixture due to some Pd particles present was filtered prior to injection onto the semipreparative HPLC column. Several different filters (eg, membrane frits) were employed for this purpose but resulted in blocking. To overcome complications caused by small filter pores, filter frit in a syringe (empty SPE column with frit) used by Onega and coworkers^11^ was applied. While this eliminated the majority of Pd‐particles present in the reaction mixture, it led to varying amounts of air in the lines between V47/V25 and the HPLC syringe. To avoid this, the SPE column with frit was prefilled with the eluent and the loading onto HPLC loop achieved in two stages: firstly, half of the eluent was withdrawn and discarded, which was followed by the insertion of the reactor needle back into the reaction solution and second withdrawal of both the crude mixture and the remaining eluent.

**Figure 3:**
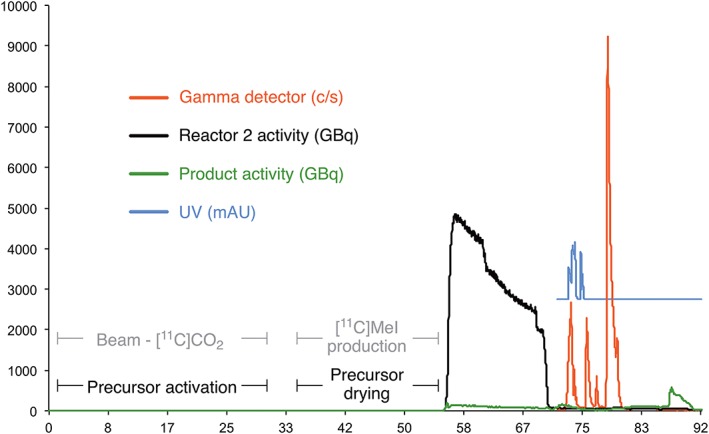
Ultraviolet and radio traces for the optimized radiosynthesis of [^11^C]UCB‐J on Synthra RNPlus module. Note that for clarity, Ultraviolet (UV) trace is increased by threefold magnitude

Following closely these requirements, conversions of up to 90% were reproducibly achieved. The radiosynthesis was successfully programmed, and typical automated profile is depicted in Figure 3. The total synthesis time from the end of [^11^C]CH_3_I production was ~35 minutes. The irradiation and the [^11^C]CH_3_I typically required ~55 minutes. Typically starting between 10 and 16 GBq of [^11^C]CH_3_I, between 0.8 and 1.4 GBq of [^11^C]UCB‐J was produced in the customer vial as a 10% EtOH solution in saline and with molar activities of 20 to 100 GBq/μmol. The nondecay corrected radiochemical yield was 11 ± 1% (n = 7) and if decay corrected 35 ± 4% (n = 7). The QC analysis of the produced [^11^C]UCB‐J indicated that the product conforms to cGMP conditions as demonstrated by the summarized QC data in Table 3.

**Table 3:** Summary of the specifications used for the cGMP radiosynthesis of [^11^C]UCB‐J for multi dose vials

QC Test (Parameter/Method)	Release Criteria	Production 1	Production 2	Production 3
Appearance	Clear colourless solution	Confronts	Confronts	Confronts
pH	4.5‐8.5	6.4	6.1	6.1
HPLC system suitability test	Retention time difference less than 8 s in two injections of reference solution	pass	pass	pass
Radiochemical identity	Retention time of [^11^C]UCB‐J corresponds to that of reference UCB‐J standard, corrected for the dead‐volume between UV and radio‐detector	pass	pass	pass
Chemical amount of UCB‐J	≤10 μg/dose	8.8	8.6	6.8
Chemical amount of desmethyl UCB‐J	≤1.5 μg/dose	0.97	0.59	0.6
Chemical amount of unidentified impurity	≤1.5 μg/dose	1.44	1.38	1.00
Total unidentified impurity	≤3.0 μg/dose	2.40	2.29	2.00
Radiochemical purity of [^11^C]UCB‐J	≥95%	100	100	100
Molar activity (GBq/μmol)	N/A	33.0	23.4	32.1
Strength (MBq in 0.1 mL)	N/A	9.1	6.3	6.8
Bacetiral endotoxins (postrelease test)	≤175 EU/dose (≤17.5 EU/mL)	≤2.5 EU/mL	≤2.5 EU/mL	≤2.5 EU/mL
Filter integrity (postrelease test)	Bubble point ≥46 psi (3.17 bar)	3.8 bar	3.7 bar	3.8 bar
Sterility (postrelease test)	Pass test	pass	pass	pass
Residual solvent DMF (postrelease test)	≤0.88 μg/day (≤880 ppm)	ND	ND	1.5
Formulation solvent EtOH (postrelease test)	≤10%	7.7%	9.8%	10%

*Note.* The limits are per injected dose. The desmethyl UCB‐J competes with UCB‐J for the binding site for which reason the specification for desmethyl UCB‐J is stringent. The unidentified impurities include all impurities in the final formulated product, and they were calculated using 258 nm wavelength absorbance response.

## 4 CONCLUSIONS

In summary, reproducible radiosynthesis of [^11^C]UCB‐J using the Synthra RNPlus automated synthesizer has been achieved with RCY at end of synthesis comparable to that reported and in compliance with the cGMP regulations. Several modifications to both the reaction conditions (eg, preactivation of the precursor) and the Synthra RNPlus module (eg, external delivery of [^11^C]CH_3_I) were required to allow for a reliable production protocol and led to identification of critical reaction parameters. This in turn allows for the implementation of the described production protocol in other clinical PET centres for routine manufacturing of [^11^C]UCB‐J.

## FUNDING SOURCES

Funding from National Institute for Health Research (NIHR) Cambridge Biomedical Research Centre, Medical Research Council grant MR/K02308X/1 is acknowledged.

## AUTHOR CONTRIBUTIONS

The manuscript was written through contributions of all authors. All authors have given approval to the final version of the manuscript.

## BRIEFS

An automated radiosynthesis of [^11^C]UCB‐J using the Synthra RNPlus platform is described.

## SYNOPSIS

The radiosynthesis of [^11^C]UCB‐J on an automated synthesizer Synthra RNPlus has been described starting from the trifluoroborate precursor and using carbon‐11 labelled CH_3_I as radiolabelling synthon.

## Supporting information


**Table S1**. Amounts and volumes of UCB‐J precursor **3** and HCl/MeOH for hydrolysis under unlabeled conditions
