# Supplementary material for: Automated radiosynthesis of [11C]UCB‐J for imaging synaptic density by positron emission tomography
Source: J Labelled Comp Radiopharm. 2020 Feb 6;63(3):151–8. doi: 10.1002/jlcr.3828 (PMC7155065; doi:10.1002/jlcr.3828)
Supplement: Supplementary file 1 — Table S1. Amounts and volumes of UCB‐J precursor 3 and HCl/MeOH for hydrolysis under unlabeled conditions [file JLCR-63-151-s001.pdf]

Supporting information: CONTENTS

Automated Radiosynthesis of [^{11}C]UCB-J for Imaging Synaptic Density by PET

Selena Milicevic Sephton,* Tunde Miklovicz, Joseph J. Russell, Aniruddha Doke, Lei Li, Istvan Boros, and Franklin I. Aigbirhio.

Experimental procedures S2-S4

General techniques S2
Radiosynthesis/QC of [^{11}C]UCB-J S2-S4
LCMS unlabeled experiments S4

LCMS Data S5

Semi-preparative HPLC Data S6

Analytical HPLC Data S7/S8

Experimental procedures

General techniques: All anhydrous solvents were purchased from Aldrich, Alfa Aesar or Acros and used as received unless otherwise noted. Water for injection was purchased from Braun and sodium chloride 0.9% w/v injection BP from Kent Pharmaceuticals. Water for cleaning of the synthesizer was Millipore (18.2 MΩ) and water used for the radiosynthesis was BP grade. The radiolabelling precursor as well as the reference material were obtained from UCB, Belgium. The Sep-Pak® Classic C18 cartridges were purchased from Waters and the empty 3 mL SPE columns with frits Chromabond from Macherey-Nagel. Ethanol for injection was purchased from Merck.
Radiochemical yields for the product were based on [^{11}C]CH_3I and decay corrected unless otherwise stated.

Radiosynthesis of [^{11}C]UCB-J on Synthra RNPlus:

Prior to each production the automated synthesizer was cleaned using H_2O and absolute EtOH and acetone. The [^{11}C]CH_3I delivery line was tested for flow and leak tightness. The GE MeI module was pre-conditioned (heating followed by the cooling of the Ni- and porapak ovens) using automated PREPARATION stage of the module.

The automated Synthra RNPlus set up was as follows (see Figure 2):

A3: 0.3 mL DMF:H_2O 8:1 v/v
B3: 1.6 mL 1M aq HCl
C1: 13 mL NaCl 0.9% BP
C2: 1.4 mL absolute EtOH
C3: 15 mL H_2O for injection
SPE: 50 mL H_2O for injection
C18: Classic cartridge, conditioned with 5 mL EtOH, 10 mL H_2O, then 2 mL air
Filter frit: filled with 3.2 mL HPLC eluent, placed between V47 and V25
HPLC: Semi-preparative column Gemini-NX C18 250x10 mm, 5 μm, 100 Å, eluting with 65% 100 mM ammonium formate pH 9.2 and 35% MeCN; flow 8 mL/min, λ = 254 nm
REACTOR 1: 1.6-1.7 mg (*R*)-trifluoro(4-((2-oxo-4-(3,4,5-trifluorophenyl)pyrrolidin-1-yl)methyl)pyridin-1-ium-3-yl)borate (UCB-J precursor) in 54 μL MeOH and 22 μL 1M aq HCl
REACTOR 2: 28 μL potassium carbonate aqueous solution (4-5 mg in 280 μL H_2O); 244 μL bis(dibenzylideneacetone)palladium(0) solution in anhydrous DMF (1.0-1.5 mg Pd(dba)$_2$ in 488 μL DMF); 200 μL tris(*o*-tolyl)phosphine solution in anhydrous DMF (2.0-2.5 mg P(*o*-Tol)$_3$ in 500 μL DMF) and 500 μL anhydrous DMF.
PRECURSOR ACTIVATION: Precursor activation is a step which commences at the same time as the cyclotron target bombardment starts and precedes the oxidative insertion of Pd to carbon-halide bond. The activation of the precursor is achieved by placing the precursor into the reactor 1, adding MeOH and HCl and stirring the mixture over 30 min at ambient temperature. The mixture is then dried in the stream of He and under vacuum and finally only vacuum after which it is re-dissolved in DMF:H_2O (0.3 mL, 8:1 v/v) and transferred from reactor 1 to reactor 2.

[^{11}C]CO_2 is produced *via* the nuclear reaction ^{14}N(p,α)^{11}C using GE PETtrace cyclotron from 0.5% O_2 enriched N_2. Typically bombardment was performed at 35 μAh over 40-45 min. The produced [^{11}C]CO_2 was then converted to [^{11}C]CH_3I using a GE MeI module *via* reduction to

[^{11}C]CH_4 using a Shimalite nickel catalyst/column followed by condensation reaction of [^{11}C]CH_4 with I_2 at 720 °C during the recirculation after which [^{11}C]CH_3I is trapped on the Porapak and released upon heating. The produced [^{11}C]CH_3I was received in the reactor 2 of the Synthra RNPlus automated module and bubbled through the mixture containing base, ligand and catalyst solution and DMF (see **REACTOR 2**) at –15 °C for 4-7 min during the number of counts per second in the reactor 2 peaked. The reaction mixture was then heated to 30 °C for 3 min to allow for oxidative insertion of Pd to carbon-halogen bond to occur. The activated precursor was then added (see **PRECURSOR ACTIVATION**) as DMF:H_2O solution and the reaction mixture was allowed to heat to 100 °C over 5 min. After this time the reaction mixture is allowed to cool to 30 °C and then quenched with HCl from B3 and loaded onto the HPLC loop *via* the syringe and by passing the mixture through the SPE column with frit containing eluent. The semi-preparative purification finally yields desired [^{11}C]UCB-J which is collected in the SPE flask containing H_2O and then trapped on the C18 Classic cartridge. The product is washed with H_2O and then eluted with EtOH and formulated as a saline solution and dispensed in the grade A isolator by passing the mixture through the sterile filter.

QC analysis: Prerelease QC of [^{11}C]UCB-J took approximately 30 minutes and was performed as described previously.[12] Release criteria and the results of three typical production experiments are summarized in Table 2. The pH of the product was determined using Seven Excellence pH meter and the visual check was performed behind a lead-shielded window. Chemical and radiochemical identity and purity were analysed by analytical radio-HPLC on the ThermoFisher Ultimate 3000 HPLC system using Waters XBridge C18 reverse phase column (3.5 μm, 100x3.0 mm) eluting with 30% MeCN in 10 mM sodium phosphate at 0.8 mL/min. The HPLC system was equipped with an UV detector (λ = 258 nm) and EG&G Ortec Amplifier 0.70 kV radioactivity detector. The radiochemical purity was determined as the percentage of the [^{11}C]UCB-J peak on the radio-chromatogram. Radiochemical identity was confirmed and assessed by comparison of the retention time of the [^{11}C]UCB-J radioactive peak to that of the UCB-J reference standard peak in the co-injection sample. The chemical amount of [^{11}C]UCB-J and cold impurities was determined by quantification and relative comparison of the corresponding UV absorbance peaks of the QC sample and reference standards of known concentration. The acceptance limit of 10 μg for UCB-J in the injection dose was established based on the selected animal toxicity study taking into account guideline on setting permitted daily exposure (PDE) by European Medical Agency (EMA). The maximal allowable injection volume (V_{max}) of radiotracers produced at the Wolfson Brain Imaging Centre (WBIC) is by default limited to 10 mL and was calculated based on the chemical amount of [^{11}C]UCB-J and cold impurities relative to their specification V_{max} = 10 μg/specification of [^{11}C]UCB-J found in a QC sample (μg/mL). Molar activity (A_m) was calculated as the ratio of radioactivity in GBq and the amount of UCB-J (μmol) at the time of anticipated application (20 min after the EOS). The integrity of the Acrodisc Supor Membrane filter (0.2 μm, 25 mm) was tested using the filter integrity (i.e., bubble point) test. Sterility, bacterial endotoxins, radionuclidic identity and residual solvent analysis of the product were assessed as the post-release tests. Endotoxin content in [^{11}C]UCB-J doses was measured with the Charles River Laboratories Endosafe Portable Test System (PTS). Residual solvent analysis was performed on Thermo Focus Gas Chromatograph equipped with the Thermo column (6′x1.8′x0.85″) and a flame ionization detector (FID). Radionuclidic identity was determined by measuring [^{11}C]UCB-J activities in triplicate at different time points (5 min intervals) using Veenstra VDC-505 radioisotope dose calibrator and the half-life was calculated as per the following: $t_{1/2} = -\ln 2 \cdot ((t_2 - t_1)/\ln(A_2/A_1))$,

whereby t_1 and t_2 are two different times and A_1 and A_2 are radioactivity measured at times t_1 and t_2, respectively.

LCMS unlabeled experiments - general

At ambient temperature and open to air, a round bottom flask was charged with UCB-J precursor and specified amount of MeOH was added followed by hydrochloric acid (See Table S1) and the reaction mixture was allowed to stir for a minimum of 30 min after which time LCMS aliquote was taken and diluted with 9:1 MeCN:H_2O and analysed.

Table S1. Amounts and volumes of UCB-J precursor **3** and HCl/MeOH for hydrolysis under unlabeled conditions

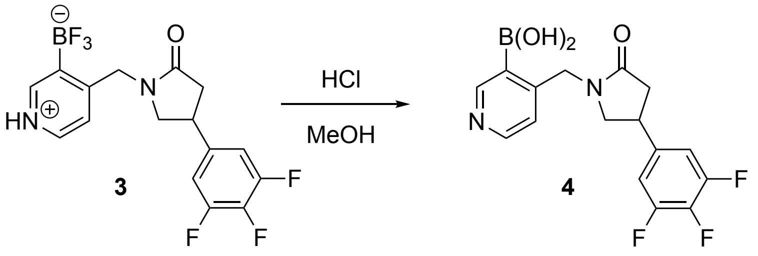

Entry	UCB-J precursor (mg)	HCl source	Conc. HCl (M)	HCl (mL)	MeOH (mL)	LCMS Conversion (%)
1	2.0	HCl in dioxane	4.00	0.25	2.00	0
2	3.0	HCl in MeOH	1.25	0.50	3.00	24
3	1.9	Aqueous HCl	1.00	0.50	2.00	39[a]
4	1.8	Aqueous HCl	1.00	0.02	0.05	0
5	1.8	Aqueous HCl	1.00	0.05	0.50	38[b]

[a] Aq. HCl added in 2x0.25 mL portions; [b] Reaction performed with the crude material

LCMS data for the UCB-J precursor activation under unlabelled conditions

Openlynx Report - Selena_M_Sephton Page 1
Sample: 1 Vial:2:39 ID:SDM_690CR
File:SDM_690CR Date:24-Apr-2018 Time:15:11:16
Method:C:\MassLynx\Generic_Pos_Neg.olp

Printed: Thu May 17 17:08:10 2018

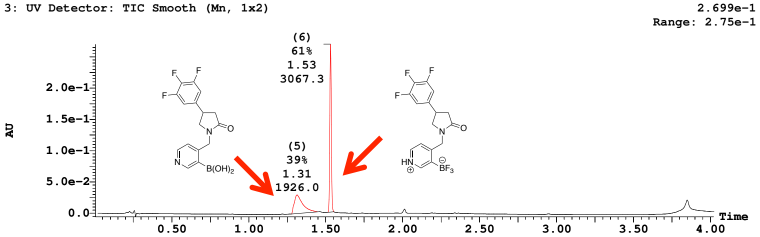

Peak Number	Compound	Time	Area %Total	Mass Found
5		1.31	38.57	
6		1.53	61.43	

Peak ID	Compound	Time	Mass Found
5		1.33	

Peak ID	Compound	Time	Mass Found
6		1.54	

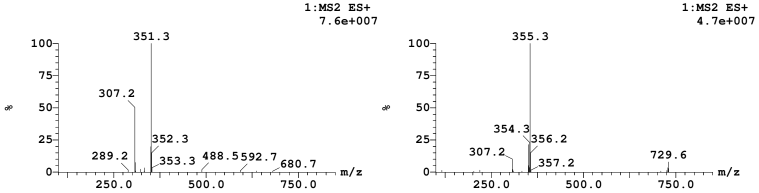

Openlynx Report - Selena_M_Sephton Page 1
Sample: 1 Vial:2:45 ID:SDM_693CR
File:SDM_693CR Date:25-Apr-2018 Time:15:13:53
Method:C:\MassLynx\Generic_Pos_Neg.olp

Printed: Thu May 17 17:10:58 2018

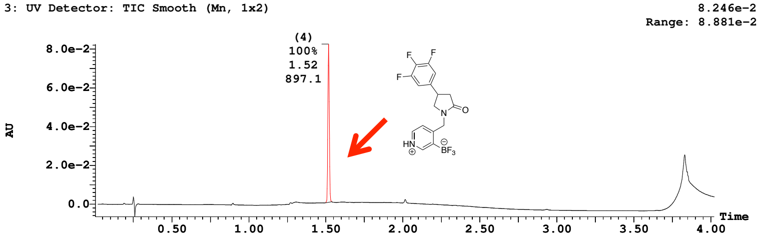

Peak Number	Compound	Time	Area %Total	Mass Found
4		1.52	100.00	

Peak ID	Compound	Time	Mass Found
2		1.29	

Peak ID	Compound	Time	Mass Found
3		1.32	

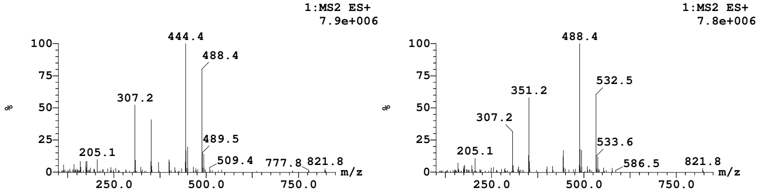

The reaction done with 1.9 mg of precursor
in 0.5 mL 1 M aq. HCl and 2 mL MeOH

Mixture soluble

The reaction done with 1.9 mg of precursor
In 20 μL 1 M aq. HCl and 50 μL MeOH

Mixture cloudy/heterogeneous

Radio chromatograph of a typical semi-preparative [^{11}C]UCB-J HPLC purification

Tuesday, 30.07.2019	SynthraView Report	UNIVERSITY OF CAMBRIDGE Wolfson Brain Imaging Centre

Filename : UCB_J_Automation1608_09.07.2019_08h46min_.rfp
Method : UCB_J_Automation1608.tcf
Synthesis started : Tuesday, 09.07.2019 , 08:48:45
Additional Information :

Chromatogram Gamma Detector

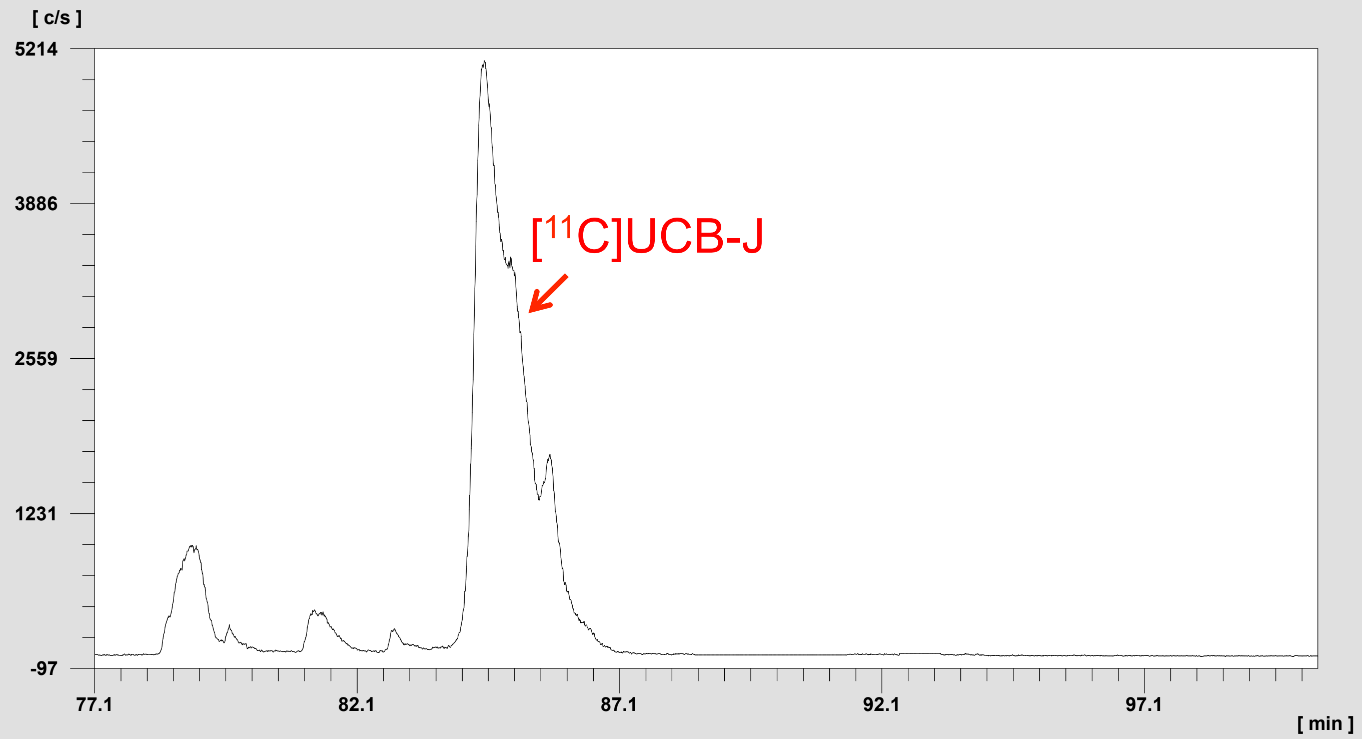

UV and Radio chromatographs of a typical analytical [^{11}C]UCB-J HPLC

Chromatogram and Results

Injection Details

Injection Name:	**SAMPLE**	*Run Time (min):*	**10.00**
Current Logon:	**sms96**	*Injection Volume:*	**20.00**
Last Modifed By:	**sms96**	*Channel:*	**RadDet**
Injection Type:	**Unspiked**	*Wavelength:*	**254.0**
Instrument Method:	**Prelim UCBJ Dev Method**	*Bandwidth:*	**n.a.**
Processing Method:	**UCBJ Prelim Meth Dev**	*Last Modified Time:*	**28/08/18 18:42**
Injection Date/Time:	**28/Aug/18 18:29**	*Print Time:*	**06/09/18 17:47**

Chromatogram

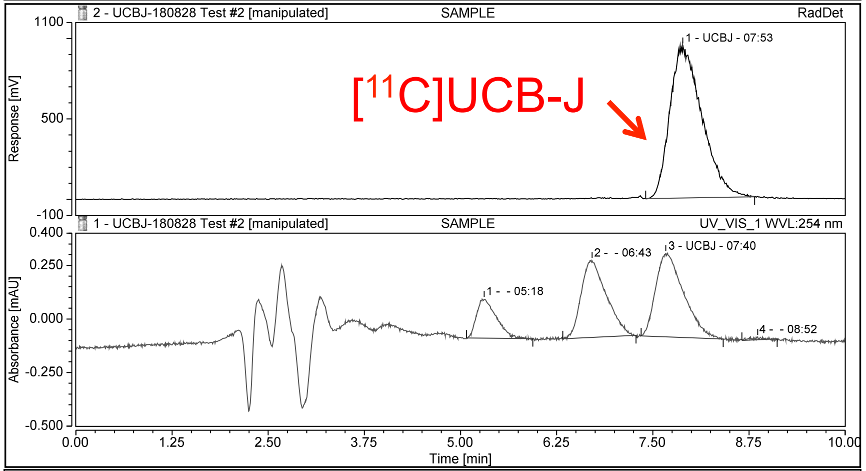

Integration Results

No.	Peak Name	Retention Time min	Area mV*min	Height mV	Relative Area %	Relative Height %	Amount
n.a.	Mel	n.a.	n.a.	n.a.	n.a.	n.a.	n.a.
1	UCBJ	7.890	458.423	948.906	100.00	100.00	n.a.
**Total:**			**458.423**	**948.906**	**100.00**	**100.00**	

Formulated solution

Chromatogram and Results

Injection Details

Injection Name:	**SPIKE**	*Run Time (min):*	**10.00**
Current Logon:	**sms96**	*Injection Volume:*	**20.00**
Last Modifed By:	**sms96**	*Channel:*	**RadDet**
Injection Type:	**Spiked**	*Wavelength:*	**254.0**
Instrument Method:	**Prelim UCBJ Dev Method**	*Bandwidth:*	**n.a.**
Processing Method:	**UCBJ Prelim Meth Dev**	*Last Modified Time:*	**28/08/18 18:52**
Injection Date/Time:	**28/Aug/18 18:41**	*Print Time:*	**06/09/18 17:47**

Chromatogram

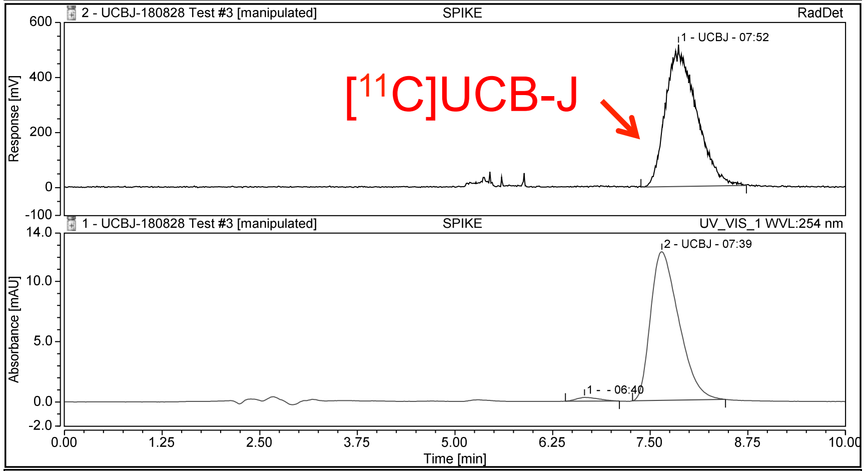

Integration Results

No.	Peak Name	Retention Time min	Area mV*min	Height mV	Relative Area %	Relative Height %	Amount
n.a.	Mel	n.a.	n.a.	n.a.	n.a.	n.a.	n.a.
1	UCBJ	7.863	231.679	514.686	100.00	100.00	n.a.
**Total:**			**231.679**	**514.686**	**100.00**	**100.00**	

Co-injected sample

UV chromatograph of a QC [^{11}C]UCB-J sample containing desmethyl analogue

Chromatogram and Results

Injection Details

Injection Name:	**SAMPLE**	*Run Time (min):*	**10.00**
Vial Number:	**BE1**	*Injection Volume:*	**20.00**
Curent Login:	**jjr50**	*Channel:*	**UV_VIS_1**
Last Modified By:	**Admin**	*Wavelength:*	**258**
Instrument Method:	**UCB-J QC Method**	*Bandwidth:*	**n.a.**
Processing Method:	**Quantitative**	*Last Modified Time:*	**10/10/2018 09:55:30 +01:00**
Injection Date/Time:	**09/Oct/18 16:35**	*Print Time*	**29/10/2018 15:30:00 +00:00**

Chromatogram

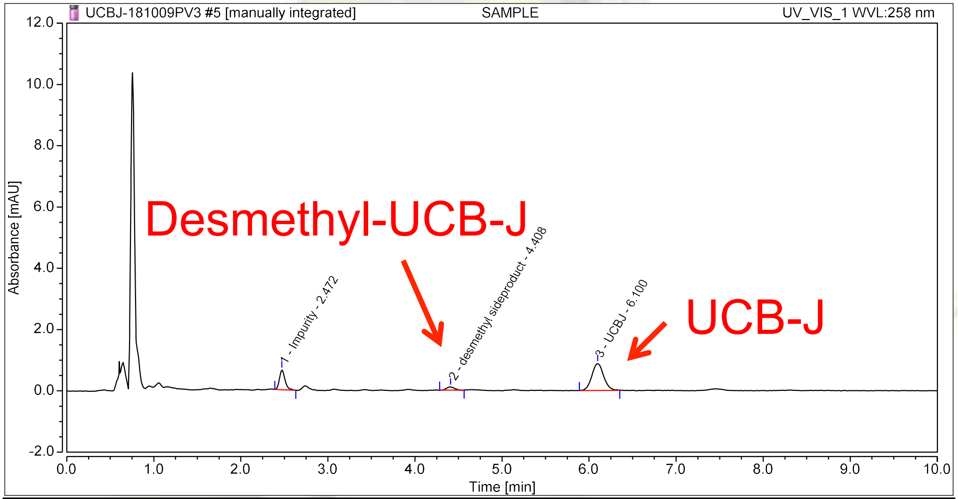

Integration Results

No.	Peak Name	Retention Time<br>min	Area<br>mAU*min	Height<br>mAU	Relative Area<br>%	Relative Height<br>%	Amount
1	Impurity	2.472	0.048	0.651	24.42	39.58	n.a.
n.a.	Precursor	n.a.	n.a.	n.a.	n.a.	n.a.	n.a.
2	desmethyl sideproduct	4.408	0.011	0.107	5.49	6.50	n.a.
3	UCBJ	6.100	0.138	0.887	70.08	53.92	n.a.
**Total:**			**0.197**	**1.646**	**100.00**	**100.00**	
